# Boosting UPR transcriptional activator XBP1 accelerates acute wound healing

**DOI:** 10.1093/pnasnexus/pgad050

**Published:** 2023-02-14

**Authors:** Jie-Mei Wang, Hainan Li, Liping Xu, Hyunbae Kim, Yining Qiu, Kezhong Zhang

**Affiliations:** Department of Pharmaceutical Sciences, Eugene Applebaum College of Pharmacy and Health Sciences, Wayne State University, 259 Mack Ave, Detroit, MI 48201, USA; Center for Molecular Medicine and Genetics, Wayne State University, 540 Canfield Street, Detroit, MI 48201, USA; Department of Biochemistry, Microbiology, and Immunology, School of Medicine, Wayne State University, 540 Canfield Street, Detroit, MI 48201, USA; Center for Molecular Medicine and Genetics, Wayne State University, 540 Canfield Street, Detroit, MI 48201, USA; Center for Molecular Medicine and Genetics, Wayne State University, 540 Canfield Street, Detroit, MI 48201, USA; Department of Pharmaceutical Sciences, Eugene Applebaum College of Pharmacy and Health Sciences, Wayne State University, 259 Mack Ave, Detroit, MI 48201, USA; Department of Pharmaceutical Sciences, Eugene Applebaum College of Pharmacy and Health Sciences, Wayne State University, 259 Mack Ave, Detroit, MI 48201, USA; Department of Pharmaceutical Sciences, Eugene Applebaum College of Pharmacy and Health Sciences, Wayne State University, 259 Mack Ave, Detroit, MI 48201, USA; Department of Biochemistry, Microbiology, and Immunology, School of Medicine, Wayne State University, 540 Canfield Street, Detroit, MI 48201, USA; Karmanos Cancer Institute, 4100 John R, Detroit, MI 48201, USA

**Keywords:** unfolded protein response, X-box-binding protein 1, wound healing, gene transfer, growth factors

## Abstract

Patients’ suffering from large or deep wounds caused by traumatic and/or thermal injuries have significantly lower chances of recapitulating lost skin function through natural healing. We tested whether enhanced unfolded protein response (UPR) by expression of a UPR transcriptional activator, X-box-binding protein 1 (XBP1) can significantly promote wound repair through stimulating growth factor production and promoting angiogenesis. In mouse models of a second-degree thermal wound, a full-thickness traumatic wound, and a full-thickness diabetic wound, the topical gene transfer of the activated form of XBP1 (spliced XBP1, XBP1s) can significantly enhance re-epithelialization and increase angiogenesis, leading to rapid, nearly complete wound closure with intact regenerated epidermis and dermis. Overexpression of XBP1s stimulated the transcription of growth factors in fibroblasts critical to proliferation and remodeling during wound repair, including platelet-derived growth factor BB, basic fibroblast growth factor, and transforming growth factor beta 3. Meanwhile, the overexpression of XBP1s boosted the migration and tube formation of dermal microvascular endothelial cells in vitro. Our functional and mechanistic investigations of XBP1-mediated regulation of wound healing processes provide novel insights into the previously undermined physiological role of the UPR in skin injuries. The finding opens an avenue to developing potential XBP1-based therapeutic strategies in clinical wound care protocols.

## Introduction

Skin wound repair needs an orchestra of multiple molecular and cellular components in three sequential but overlapping phases, inflammation, proliferation, and remodeling (1). In adults, skin wounds caused by traumatic and/or thermal injuries, particularly large or deep injuries, often fail to recapitulate skin structure and heal by scar, many of which can cause cosmetic or functional defects. When skin wounds occur in patients with diabetes or atherosclerosis, these wounds can easily progress to nonhealing ulcers despite standard wound care protocols (2). Therefore, there is an unmet need for effective regimens conducive to a favorable microenvironment for wound healing.

The high demand for protein folding and clearance of malfunctional proteins and the increase in new protein synthesis in wound repair and regeneration increases the burden on the endoplasmic reticulum (ER), disrupting ER homeostasis and causing ER stress (3). The ER stress response, or unfolded protein response (UPR), classically encompasses the activation of three separate ER transmembrane stress sensors, with the inositol-requiring enzyme 1α (IRE1α)/X-box-binding protein 1 (XBP1) pathway being the most conserved UPR pathway among species (4). Once activated, IRE1α cleaves a 26-base pair segment from XBP1 transcripts and produces the active, spliced isoform of XBP1 (XBP1s), functioning as a transcriptional factor to induce multiple genes involved in the restoration of ER homeostasis (4). XBP1 is known to exert substantial effects in augmenting ER protein folding capacity and remodeling the secretory pathway (4, 5). In addition, XBP1 might carry out distinct roles in vascular development in the fetal stage (6). However, the potential roles of XBP1 in regulating the adult tissue repair process are unclear to date.

In this study, we evaluated the efficacy of XBP1 gene transfer on burn and diabetic wounds. Downstream pathways in fibroblasts and endothelial precursor cells have been explored to dissect the XBP1-induced skin repair and regeneration capacity. Our study has provided novel discoveries implicating a potentially high-effective therapeutic approach for acute burn or traumatic injury-associated wound healing through enhanced XBP1 expression.

## Results

### XBP1 improves burn wound healing in vivo

**Fig. 1.**
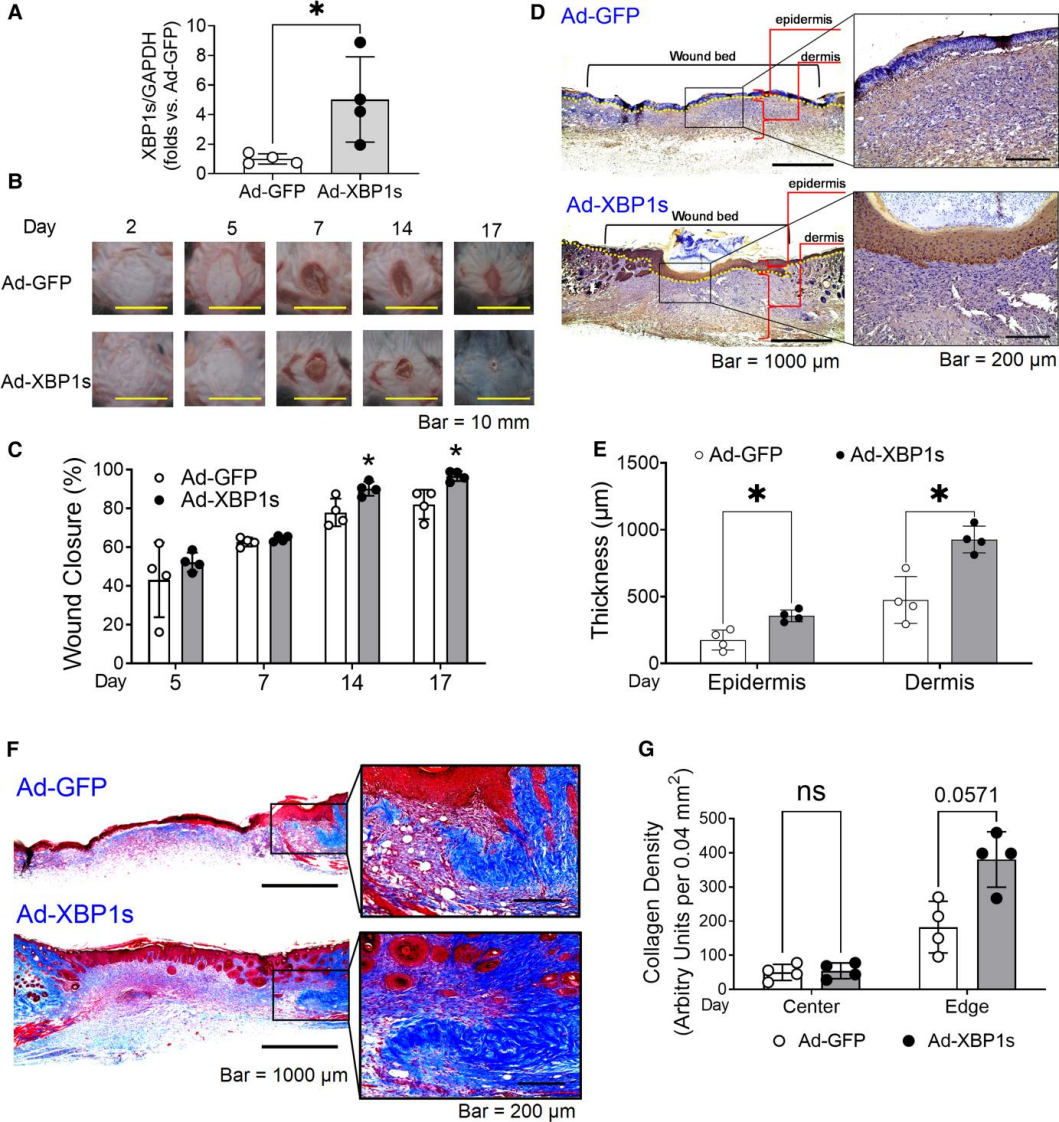
XBP1 gene transfer improves burn wound healing. A) Real-time PCR analysis of spliced XBP1 levels in wound tissue harvested at the end of the experiment. B) Burn wound images. C) Wound closure throughout healing courses. D) Keratin 14 staining (in brown color) of wound sections. The dotted lines separate the epidermis and dermis. E) The thickness of the epidermis (based on keratin 14 staining) and dermis (based on Masson's trichrome staining). F) Collagen fibers stained by Masson's trichrome. G) Collagen density within wounds. In all the figures, *n* = 4 per group, ns, not significant. *P < 0.05.

In a mouse model of the deep partial-thickness burn wound, we infected the wounds with the adenovirus carrying activated/spliced XBP1 (Ad-XBP1s) sequence 2 days after wounding, using adenovirus carrying *egfp* (Ad-GFP) as a control. The elevated mRNA levels of activated/spliced XBP1 (XBP1s) in wound tissue were confirmed by real-time PCR at the end of the experiment (Fig. 1A). Our results showed that Ad-XBP1s healed the wounds significantly faster than Ad-GFP (Fig. 1B and C), starting from day 14. At the end, wounds receiving Ad-XBP1s were considered clinically healed, while wounds receiving Ad-GFP were covered by large scabs. The keratin 14 staining suggested that XBP1s-expressing wounds processed a continuous, thick, and slightly keratinized epidermis (Fig. 1D), and a decent depth of dermis (Fig. 1E). In contrast, GFP-expressing wounds had no or partially formed epidermis and thin dermis with extensive inflammatory cells (Fig. 1D and E). The Masson's trichrome staining suggested that the collagen fibers were moderately increased (*P* = 0.0571) and better organized and corrugated structured at the wound edge of XBP1s-expressing wounds, although they were comparable in the wound center area (Fig. 1F and G). These data indicate that XPB1s is potent in promoting the regeneration of functional dermal and epidermal layers for burn-injured skin and refining the inflammation.

### XBP1 induces growth factor and cytokine expressions

**Fig. 2.**
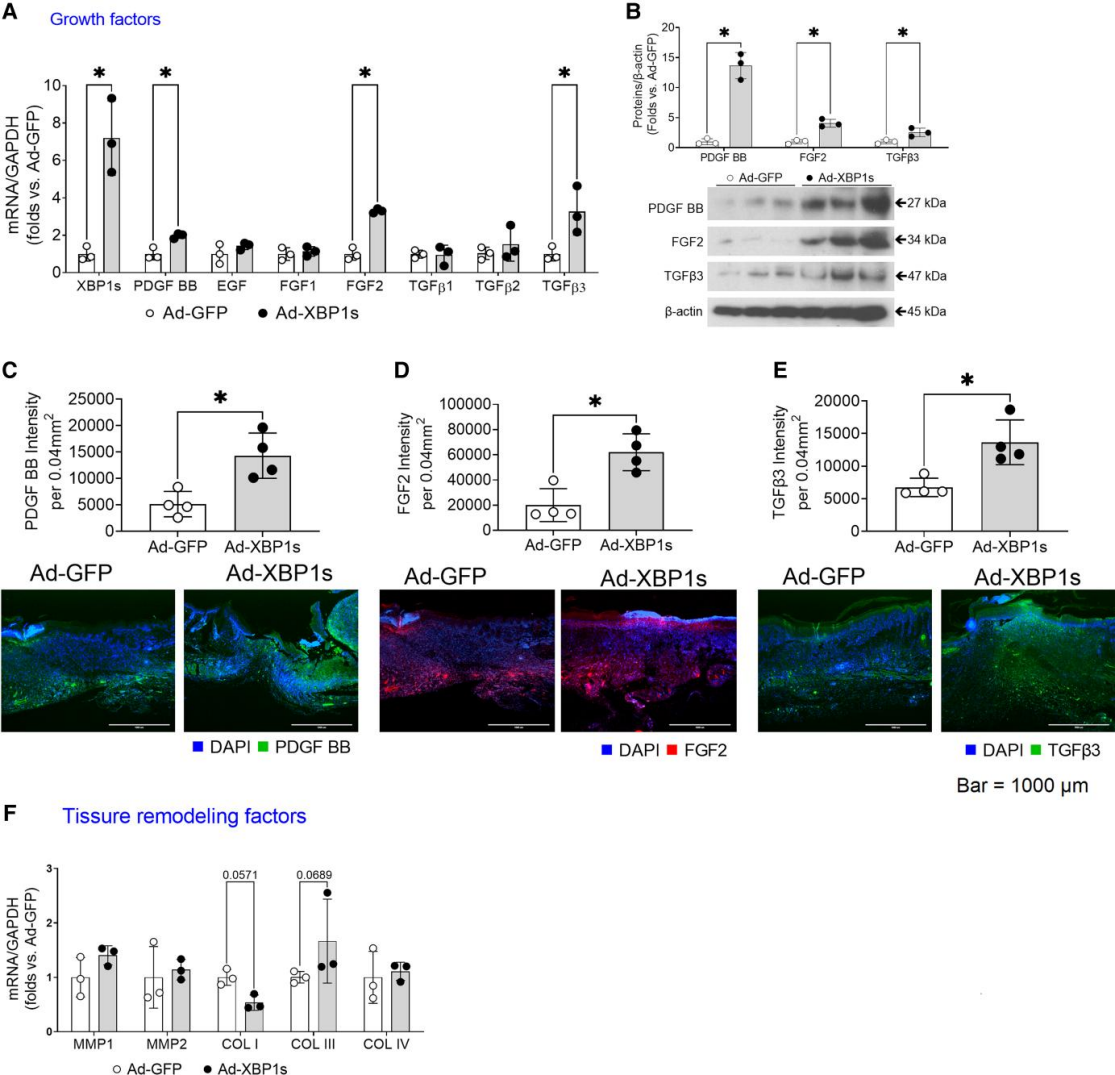
XBP1 augments mRNA expression levels of PDGF BB, FGF2, TGFβ3. A) The mRNAs coding growth factors in human embryonic kidney fibroblast/endothelial/epithelial cells expressing XBP1s or GFP controls. *n* = 3 per group. *P < 0.05. B) The protein levels of PDGF BB, FGF2, TGFβ3 were measured by Western blot. The quantifications of PDGF BB (C), FGF2 (D), and TGFβ3 (E) in burn wound samples were determined by immunofluorescent staining of the target proteins in three high-power fields randomly taken in the wound center. F) The mRNAs coding remodeling cytokines in XBP1s-expressing cells and their GFP controls. In all the figures, *n* = 3 per group, *P < 0.05.

Next, we sought to investigate whether XBP1 boosts growth factors. We overexpressed XBP1s or GFP control in human embryonic kidney cells (consisting of fibroblasts, endothelial cells, and epithelial cells). Real-time PCRs showed that mRNAs encoding the growth factors (Fig. 2A), including platelet-derived growth factor (PDGF) BB, basic fibroblast growth factor (FGF2), and transforming growth factor beta 3 (TGFβ3), were significantly elevated in XBP1s-expressing cells. The protein levels of these three growth factors were increased by XBP1s gene transfer as measured by Western blot (Fig. 2B). Furthermore, immunofluorescent staining of PDGF BB (Fig. 2C), FGF2 (Fig. 2D), and TGFβ3 (Fig. 2E) in the wound sections suggested that XBP1s gene transfer increased these growth factors in wound healing in vivo. As shown in Fig. 1F, tissue remodeling factors such as collagen type III (COL III) were moderately increased (*P* = 0.0689), while collagen type I (COL I) moderately decreased (*P* = 0.0571).

### XBP1 accelerates wound healing in healthy and diabetic animals in vivo

**Fig. 3.**
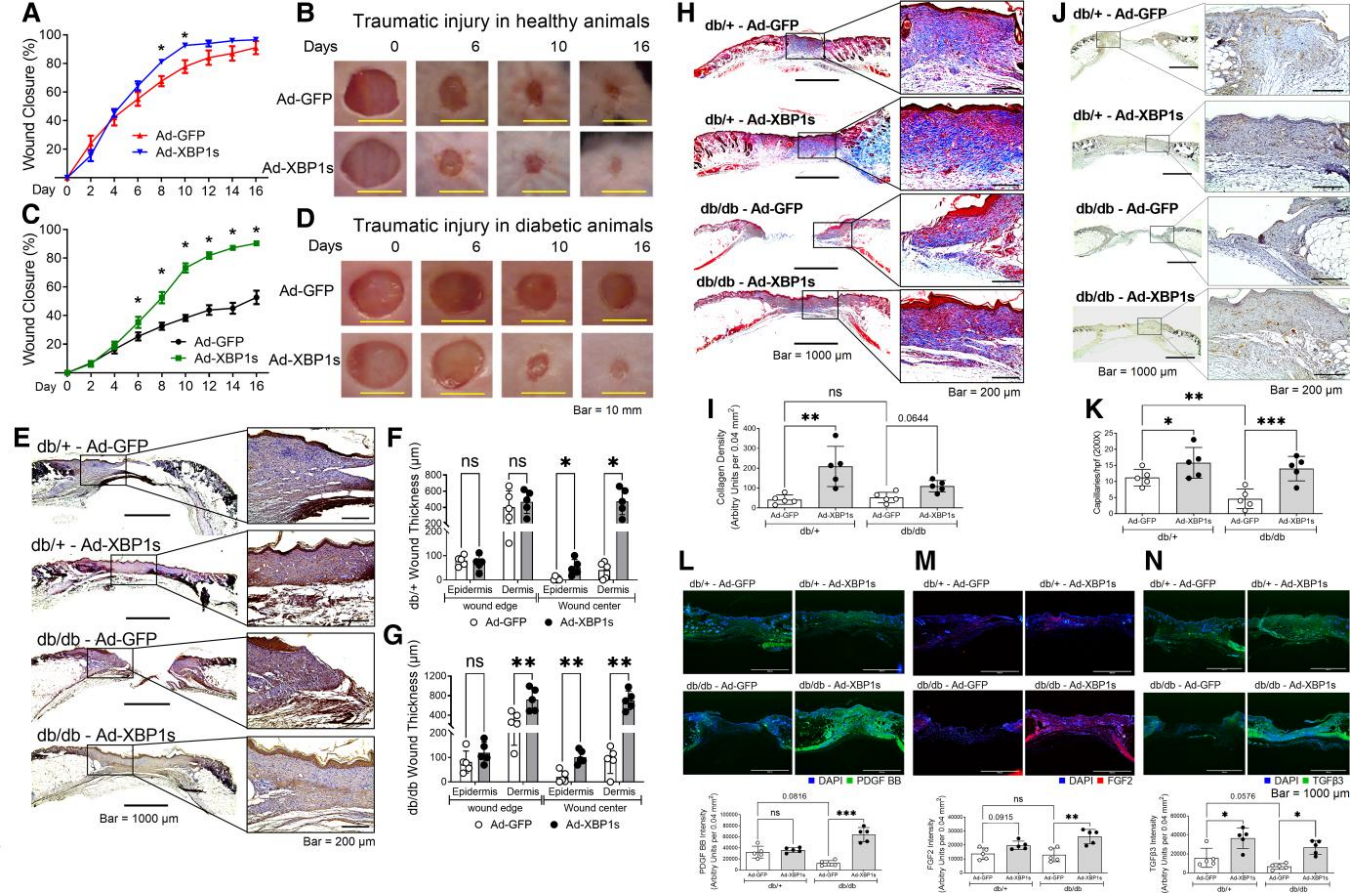
XBP1 gene transfer accelerates diabetic wound healing. A) Wound closure after topical Ad-XBP1 or Ad-GFP treatments in healthy (db/+) animals. *n* = 5, *P < 0.05 vs. db/+-Ad-GFP. B) Wound images of db/+ mice. C) Wound closure after topical Ad-XBP1 or Ad-GFP treatments in type 2 diabetic (db/db) animals. *n* = 5, *P < 0.05 vs. db/db + Ad-GFP. D) Wound images of db/db mice. E) Keratin 14 staining (in brown color) of wound sections. The epidermis and dermis thickness in healthy db/+ mice (F) and db/db mice (G) were determined. H) Masson's trichrome staining (in blue color) of wound sections. I) Quantitation of collagen fibers. J) Capillary-like structures stained by CD31 (in brown color) in the wound bed. K) Quantification of capillary density. The quantifications of PDGF BB (L), FGF2 (M), and TGFβ3 (N) in wound sections. In all the figures, *n* = 5, ns, not significant, *P < 0.05. **P < 0.01. ***P < 0.0001.

In traumatic wounds (full-thickness excisional) in either type 2 diabetic (db/db) or normal (db/+) mice, Ad-XBP1s was applied to in the wound bed immediately after wounding using Ad-GFP as control. In healthy mice (db/+), Ad-XBP1s accelerated wound healing starting from day 8 and reached full closure on day 10 when the control wounds were healed by <80% (Fig. 3A and B). More importantly, in type 2 diabetic mice (db/db), the Ad-XBP1s-treated wounds demonstrated significantly faster closure from day 2 (Fig. 3C and D). In the end, Ad-XBP1s-treated wounds were ∼80% closed, while control wounds were healed only by ∼50%. These results indicated that enhancing XBP1 accelerates tissue repair in both healthy and diabetic animals with more remarkable efficacy in diabetic animals. Keratin 14 staining suggested that XBP1s gene transfer augmented wound re-epithelialization in healthy and diabetic animals (Fig. 3E). The epidermal layers and dermal layers were thicker in XBP1s group (Fig. 3F and G). The collagen deposition was enhanced by XBP1s in wound dermal layers in healthy animals and a similar trend in diabetic animals (Fig. 3H and I). In both normal and diabetic wounds, XBP1s also stimulated capillary formation at the wound edge where the most robust angiogenesis occurred, as stained by CD31 (Fig. 3J and K). Meanwhile, we also found increased levels of PDGF BB (Fig. 3L), FGF2 (Fig. 3M), and TGFβ3 (Fig. 3N) in wound beds by immunofluorescent staining.

### XBP1 promotes angiogenesis in vitro

**Fig. 4.**
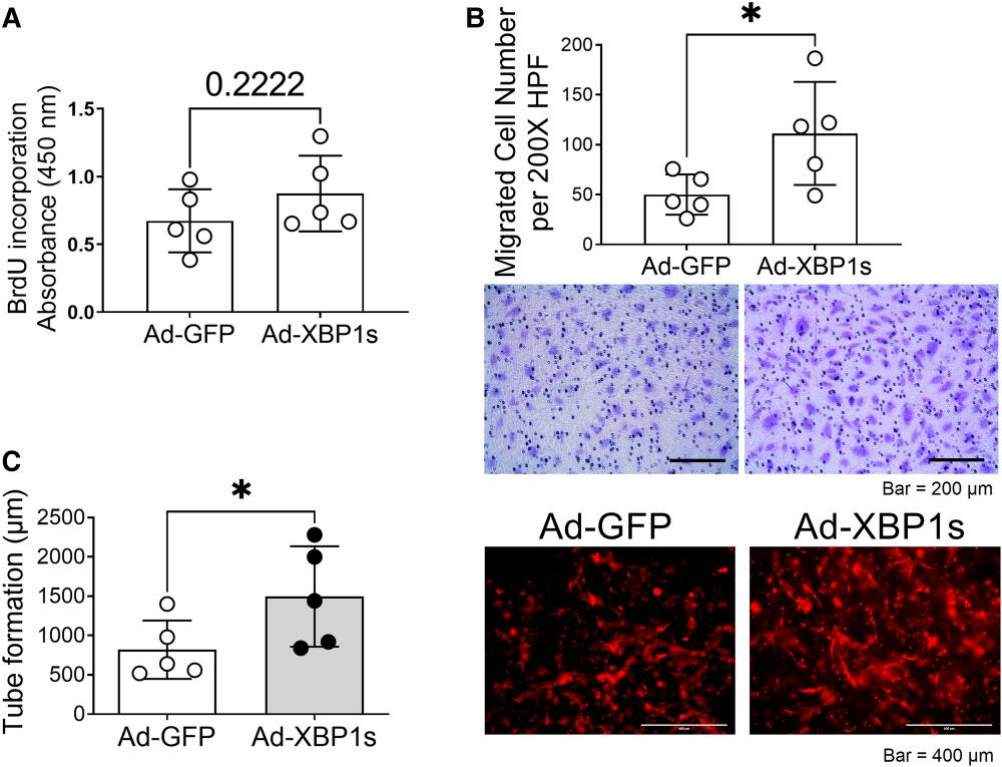
Overexpression of spliced XBP1 augments angiogenesis of HDMVECs in vitro. (A) Cell proliferation measured by BrdU incorporation. (B) Cell migration evaluated by the quantification of migrated cells in a modified Boyden chamber. (C) The 3-D tube formation in a collagen gel quantified by the accumulated tubular network length. The HDMVECs were stained by Ulex-lectin enriched at the cell surface. In all the assays, *n* = 5. *P < 0.05.

Human dermal microvascular endothelial cells (HDMVECs) were transfected with Ad-XBP1s or Ad-GFP. The functional assays showed that while their proliferation stayed comparable (Fig. 4A), HDMVECs transfected with Ad-XBP1s displayed significantly higher migration (Fig. 4B) and tube formation activity (Fig. 4C) compared with their controls, suggesting the permissive role of XBP1in angiogenesis.

## Discussion

In this study, we have obtained strong evidence that exogenous expression of the UPR transcriptional factor XBP1 boosts angiogenesis and epithelialization, leading to rapid, nearly complete wound healing in burn wounds in healthy animals or traumatic wounds in diabetic animals. Potential mechanisms involved include cytokine/growth factor expression and the augmentation of angiogenesis.

PDGF BB, FGF2, and TGFβ3 were significantly upregulated in XBP1s-expressing cells and wounds (Figs. 2C–E and 3L–N), which supports XBP1's action in modulating the proliferative phase. Angiogenesis and growth factor production is essential for diabetic wound healing. PDGF is currently the only growth factor treatment for diabetic wounds approved by FDA (7). FGF2 has been reported to contribute critically to the modulation of scar formation (8). TGFβ family members promote re-epithelialization and angiogenesis (9), with TGFβ3 uniquely inhibiting myofibroblast formation and scarring in adult wounds (10). These growth factors were chosen because of their significant roles in the skin healing process, but it is worth investigating many more factors under the control of XBP1. For example, previous reports have shown mutually positive interactions between VEGF and XPB1 splicing in various settings (6, 11), serving as a potential mechanism for improved angiogenesis in diabetic wounds. In fact, we observed an overall moderately increased response of inflammatory cytokine expression upon XBP1 expression (Supplemental Fig. S1). Among them, the increase of anti-inflammatory, pro-angiogenic IL-8 (12) reached statistical significance, and there was a trend that pro-inflammatory IL-1β was also increased (*P* = 0.165). While inflammatory cytokines are required for the wound-healing process, excessive levels of the pro-inflammatory cytokines might harm the angiogenesis or wound-healing process (13). However, our in vivo data have shown that XBP1s facilitates the healing process and promotes wound angiogenesis. These observations support that XBP1s stimulates the production of select anti-inflammatory cytokines while not acting as a negative factor in angiogenesis.

The healing outcome of burn wounds largely relies on scar formation, matrix deposition, and reorganization, which are critical events in wound proliferation and remodeling. Our histological analyses suggested that XBP1s gene transfer increased collagen fibers in dermal layers of the wounds (Fig. 1F and G), indicating enhanced skin strength, therefore, potentially improved wound quality. However, there was a concern that prolonged or extensive XBP1 activation might increase hypertrophic scar formation because a previous study found that the inhibition of XBP1 upstream activator IRE1α decreased scar formation in a mouse excisional wound model (14). The collagen turnover in dermal layers can occur at a rapid pace. While Col I is always the primary collagen type, in a healing wound, Col III is the first to be synthesized in the early stage and is replaced by Col I (15). Collagen in post-burn hypertrophic scarring consists primarily of Col I and less Col III, compared with the uninjured dermis (15). Our in vitro data suggested that XBP1 activation induced a moderate decrease in Col I but an increase in Col III (Fig. 2F), suggesting that Col III might be the dominant type in the XBP1s-treated wounds. In addition, persistent activation of XBP1 may trigger carcinogenic responses due to boosting growth factors. While it is beyond the scope of the current study to examine the long-term safety concerns of XBP1 gene transfer, the abovementioned observations have constituted a valid concern needed to be addressed in the future.

In conclusion, our study uncovered a previously undetermined role of the key UPR activator, XBP1, in promoting re-epithelialization and facilitating tissue repair and regeneration in acute burn, traumatic, and diabetic wounds. Applying the XBP1-based treatment protocol might have a high potential to be translated into the clinical arena.

## Materials and methods

### Animals

Male C57BL/6 mice at the age of 12 weeks, type 2 diabetic mice (BKS.db, age of 12 weeks), and their age- and gender-matched nondiabetic healthy littermates (db/+) were purchased from Jackson Laboratory (Bar Harbor, ME, USA). All animal procedures were performed according to Wayne State University Institutional Animal Care and Use Committee (IACUC) guidelines.

### Gene therapy for burn wound healing in vivo

Wounds of a second-degree deep contact thermal injury were created to dorsal skin by placing a 1.2-cm diameter stainless steel rod (Fisher Scientific) rod heated to 95°C and applied to the prepped area for 10 s, causing a wound of ∼3.0 cm^2^ (16). On day 2, a square of 1.5 by 1.5 cm per area was injected with 2 × 10^8^ particle forming units (PFUs) of either Ad-XBP1s (kindly provided by Dr Umut Ozcan from Harvard University) or Ad-GFP (Purchased from Vector Biolabs) solved in 100 μL PBS (17). The XBP1s gene transfer was expected to be effective on day 4 when the wounds were at the peak of denatured protein cleavage and the beginning of new tissue generation. Wound closure rates were calculated as Percentage Closed (*y*%) = [(Area on Day_0_ – Open Area on Day*_x_*)/Area on Day_0_] × 100.

### Gene therapy for diabetic wound healing in vivo

A full-thickness excisional wound was created on the dorsal skin of the animal using a 6-mm punch biopsy (18). Immediately after wounding, 2 × 10^8^ PFU of Ad-XBP1s or Ad-GFP loaded was injected into the wound edge (19). Wounds were covered with oxygen-permeable wound dressing (Tegaderm) and changed every 2 days. Wound closure rates were calculated as described above (18).

### Human embryonic kidney cell culture and adenovirus infection

Human embryonic kidney cells enriched with fibroblastic, endothelial, and epithelial cells, were transfected with Ad-XBP1s or Ad-GFP at an MOI of 500 for 48 h, as described previously (20). Total RNA was extracted to perform qRT-PCR analyses for growth factor/cytokine expressions.

### HDMVEC culture, adenovirus infection, and functional assays

HDMVECs were purchased from Lonza and maintained at passage 4–7. After transfected with Ad-XBP1s or Ad-GFP (500 MOI, 48 h), they were subject to in vitro functional assays: proliferation measured by BrdU incorporation; migration measured by modified Boyden chamber; and 3-dimensional *Ulex*-lectin stained tubular formation in collagen gel.

### Statistics

All values are expressed as mean ± SD. The statistical significance of differences between the two groups was determined using the Mann–Whitney *U* test. For the in vivo wound closure data, 2-way repeated-measures ANOVA followed by *Bonferroni post-hot* testing was used to compare both differences between treatments and time courses (18). In all tests, *P* < 0.05 was considered statistically significant. The statistical analyses were performed using GraphPad Prism 9 (GraphPad Software).

The extended methods can be found in Supplementary Information.

## Supplementary Material

pgad050_Supplementary_Data

## Data Availability

The datasets generated and analyzed during the current study are included in this manuscript and the supplemental materials.
