# Supplementary material for: Boosting UPR transcriptional activator XBP1 accelerates acute wound healing
Source: PNAS Nexus. 2023 Feb 14;2(3):pgad050. doi: 10.1093/pnasnexus/pgad050 (PMC10028334; doi:10.1093/pnasnexus/pgad050)
Supplement: pgad050_Supplementary_Data [file pgad050_supplementary_data.docx]

**
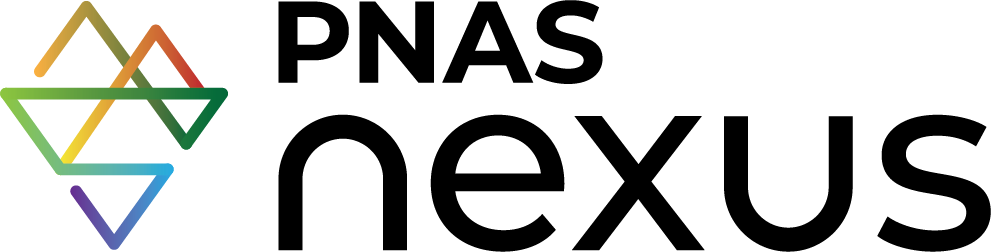
**

**Supplementary Information for**

Boosting UPR Transcriptional Activator XBP1 Accelerates Acute Wound Healing

Jie-Mei Wang, MD, PhD ^1,2,4*^; Hainan Li, MS^2^; Liping Xu, BS^2^, Hyunbae Kim, PhD^1^; Yining Qiu, MD, PhD^1^; Kezhong Zhang, PhD ^1,3,4,*^

^1^Center for Molecular Medicine and Genetics, ^2^Department of Pharmaceutical Sciences, Eugene Applebaum College of Pharmacy & Health Sciences; ^3^Department of Biochemsitry, Microbiology, and Immunology, ^4^Karmanos Cancer Institute; Detroit, MI.

* Corresponding authors:

Jie-Mei Wang, MD PhD, Department of Pharmaceutical Sciences, Eugene Applebaum College of Pharmacy & Health Sciences, Wayne State University. Address: 259 Mack Ave, 3122 Applebaum Building, Detroit, MI 48201, USA. Tel: +1 313 577 1715; Fax: +1 313 577 5218; E-mail: [jiemei.wang@wayne.edu](mailto:jiemei.wang@wayne.edu)

Kezhong Zhang, PhD, Center for Molecular Medicine and Genetics; 3Department of Biochemistry, Microbiology, and Immunology; Wayne State University School of Medicine. Address: 540 E. Canfield Avenue, 3202 Scott Hall, Detroit, MI 48201, USA. Tel.: +1 313 577 2669; Fax: +1 313 577 5218; E-mail: [kzhang@med.wayne.edu](mailto:kzhang@med.wayne.edu)

**This PDF file includes:**

Extended Methods

Supplemental Figure S1.

SI References

**Supplementary Information Text**

**Extended Methods**

**Animals.** Male C57BL/6 mice at the age of 12 weeks were purchased from Jackson Laboratory (Bar Harbor, ME). Male type-2 diabetic mice (BKS.Cg-m^+/+^Lepr^db^/J, db/db, age of 10-12 weeks), and their age- and gender-matched non-diabetic healthy littermates (BKS.Cg-m^-/-^ Lepdb/^_^ lean, db/+) were purchased from the Jackson Laboratory. All animal procedures were performed according to Wayne State University Institutional Animal Care and Use Committee (IACUC) guidelines.

**Gene therapy for burn wound healing *in vivo*.** Wounds of thermal injury were created using a previously published method (2). C57BL/6 mice at the age of 10 weeks were prepped by shaving an area corresponding to the thermal injury location, sterilized, and received a deep partial-thickness (second-degree deep dermal) contact burn to the dorsal surface, causing a wound of ~3.0 cm^2^. Subcutaneous lidocaine injection was performed to give local anesthesia and to protect muscle beneath the designated area. Wounds were inflicted by placing a 1.2-cm diameter stainless steel rod (Fisher Scientific) in a water bath heated to 95 °C and applied to the prepped area for 10 seconds. The animals were given subcutaneous S.R. buprenorphine 1 mg/kg body weight 30 minutes prior to thermal insult and were administered lactated Ringer’s solution (0.1 ml/g body weight) immediately post-burn. Burn wounds were then dressed with sterile saline gauze and covered with adhesive wound dressing (3M) that is larger than the wound area. Adenovirus expressing spliced XBP1 (XBP1s) was kindly provided by Dr. Umut Ozcan (Harvard University) (3). On day 2, wound dressing was removed, and a square of 1.5 by 1.5 cm per area will be transfected with 2 × 10^8^ particle forming units (pfu) Ad-XBP1 or Ad-GFP solved in 100μL PBS (50μL injection to wound edge and 50μL injection to wound bed). After infection, the wounds were dressed as mentioned above. A standard wound care protocol was provided, including debridement of the burn under isoflurane and dressing changes every day for the first week and every other day afterward. Wound areas were recorded, digitalized, calculated with a computerized algorithm and converted to percent wound closure. Wound closure rates were calculated as Percentage Closed (y%) =[(Area on Day_0_–Open Area on Day_x_ )/Area on Day_0_]×100.

**Gene therapy for normal and diabetic wound healing *in vivo.*** Wounds were created on the dorsal skin of the mouse, as we previously described (4). Full-thickness skins were removed using a 6-mm punch biopsy without hurting the underlying muscle. For XBP1 gene transfer, 10^8^ pfu of Ad-XBP1s or Ad-GFP were pre-loaded in 40μl PBS at 4°C. The adenovirus suspension was injected onto the wound edge in the panniculus carnosus layer immediately after wounding, using a Hamilton syringe and 30½ gauge needle as described previously (5). The grouping was as follows: 1) db/+ wound with Ad-GFP; 2) db/+ wound with Ad-XBP1s; 3) db/db wound with Ad-GFP; and 4) db/db wound with Ad-XBP1s. Wounds were covered with transparent oxygen-permeable wound dressing (Bioclusive, Johnson & Johnson). The dressings were changed every other day. Wound closure rates were measured by tracing the wound area onto acetate paper. The tracings were digitized, and the areas were calculated with a computerized algorithm and converted to percent wound closure (Image J). Wound closure rates were calculated as Percentage Closed (y%) =[(Area on Day_0_–Open Area on Day_x_ )/Area on Day_0_]×100, as we described previously (4).

**Immunohistology analysis of wound tissues** In the burn wound healing experiment, the wound tissue was recovered on day 17. In the diabetic wound healing experiment, the wound tissue was recovered on day 6. Wounds and the adjacent skin were collected. Immunohistochemical staining was performed on formalin-fixed, paraffin embedded. After deparaffinization and heat retrieving, sections were blocked by 5% goat serum + 0.2% Triton X-100 solutions. Mouse Keratin 14 antibody (Invitrogen, MA511599) or platelet and endothelial cell adhesion molecule 1 (PECAM-1, CD31) (BD Pharmingen, 550274) antibody was used at 1:200 dilution overnight. Followed by 2^nd^ antibody incubation using VECTASTAN ABC kit (Vector Laboratories, PK-6100) and DAB peroxidase substrate kit (Vector Laboratories, SK-4100) according to the manufacturer’s protocol. For Masson’s trichrome staining, after deparaffinization, the sections were stained by Trichrome staining (Tyr Scientific LLC) followed by the manufacturer’s protocol. Red represents keratin and muscle fibers, blue represents collagen, light red or pink represents the cytoplasm, and dark brown or black represents the nuclei. For immunofluorescence staining, the sections were deparaffinized and retrieved in a microvan for 20 min, followed by 10% BSA +0.2% Triton X-100 solution blocking, incubated with either Rabbit TGF b3 (Invitrogen, PA599186, 1:200), or Mouse FGF 2 (Origene, TA500012, 1:100), or Rabbit PDGF BB (Abcam, ab23914, 1:250) overnight, followed by incubation with Alexa Fluor 488- or 594-conjugated secondary antibodies (mouse or rabbi, respectively), and mounted with anti-fade DAPI mounting solution. The wound images were taken under an EVOS FL Imaging System (Invitrogen). Three random high-power fields from each slide were taken in the center and the edges of the wound and the relative area of collagen was determined using Image J (National Institutes of Health, 1.53v).

**Human dermal microvascular EC (HDMVEC) culture and adenoviral transfection**

Human dermal microvascular endothelial cells (HDMVECs) from healthy donors and patients with type 2 diabetes (T2D) were purchased from Lonza and maintained in microvascular endothelial growth medium-2 (EGM-2 MV, Lonza) in 37^o^C, 5% CO_2_ (6). Donor information: healthy donors, n=5, age 58.8 ± 1.068 years, male/female = 1:4; T2D donors, n=3, age 69.67 ± 2.028 years, male/female = 2:1. All the HDMVECs underwent 4 to 7 passage cycles and were grown until they were 70-90% confluent. For transfection of cells with adenovirus, cells were seeded in six-well plates. After 24 hours, cells were transfected with Ad-XBP1s or Ad-GFP at an MOI of 500 for 48 hours, as described previously (1).

**Cell Functional Assays (3D tube formation, Migration, and Proliferation)**

Angiogenesis was evaluated by a modified 3D tube formation assay as previously described (7). For the preparation of 1 mL of collagen/media solution, 340 μL of type I rat tail collagen (Corning, 354236), 76 μL of 10 × M199 (Sigma, M0650), 136 μL serum-free DMEM, 100 μL FBS and 136 μL of DPBS were mixed on ice. The pH was adjusted to 7.2 with NaOH. 1.8 × 10^6^/mL cells were mixed for a final collagen concentration of 1.25 mg/mL. An amount of 30 μL of collagen/cell mixture was loaded onto into a 48-well culture plate. The culture media was added after 1 hour of polymerization of the collagen/cell mixture at 37 °C, 5% CO_2_. The culture media consisted of EBM-2 supplemented with all EGM-2 bullet kit components except FBS and VEGF and was supplemented with 1% FBS, 30 mg/mL VEGF-A^165^ (Peprotech, 100-20). On day 6-8, the collagen-embedded cells were fixed in 4% formaldehyde and stained with 10 ug/mL of Lectin from *Ulex europaeus*-Atto 594 conjugate (Sigma, 73873). Cell proliferation was evaluated using BrdU Cell Proliferation Assay Kit (Cell Signaling, #6813). Migration was assessed by a modified Boyden chamber assay as previously described (7). The Transwell inserts with 8-μm pore size were coated with 0.5% gelatin. 3×10^4^ cells were loaded into the upper chamber with FBS-free media. The inserts were transferred to the lower chamber in a 24-well plate containing medium with 10% FBS. The cells were incubated for 6 h at 37 °C, 5% CO_2_. Cells that had not migrated from the top of the insert membrane were removed by wet cotton swabs. Cells that migrated to the lower side of the membrane were fixed with 4% paraformaldehyde for 10 min and stained with crystal violet. The migrated cells were counted in a mean value of 5 different fields at 100X magnification for each sample under an inverted microscope (EVOS FL, Invitrogen).

**Human embryonic kidney cell culture and adenovirus infection. Human embryonic kidney cells consisting primarily fibrolasts, endothelial cells, and epithelial cells were purchased from ATCC and cultured in DMEM medium plus 10% fetal bovine serum.** For transfection of cells with adenovirus, cells were seeded in six-well plates until 80%-90% confluency. After 24 hours, the cells were transfected with Ad-XBP1s or Ad-GFP at an MOI of 500 for 48 hours, as described previously (1).

**Quantitative real-time PCR (qRT-PCR) analyses.** Total RNA from BMACs was isolated by RNeasy Mini Kit (Qiagen). For mRNA expressions, cDNA was generated from 200ng of RNA using High-Capacity cDNA Reverse Transcription Kit (Life Technologies). Quantitative real-time PCR was performed using cDNA generated from 200ng of RNA. The primers were synthesized by Integrated DNA Technologies, Inc. Amplification and detection of specific products were performed with the ABI PRISM 7500 Sequence Detection System, using *gapdh* as an internal control. Fluorescent signals were normalized to an internal reference, and the threshold cycle (C_t_) was set within the exponential phase of the PCR. The relative gene expression was calculated by comparing cycle times for each target PCR. The C_t_ value was normalized by subtracting the *gapdh* C_t_ value, which gave the ΔC_t_ value. The relative expression levels of each target between groups were then calculated using the following equation: relative gene expression = 2^-(ΔCt_treatments-ΔCt_controls)^ as we previously described (4).

**Western Blot Analysis**. Cell lysates were collected by CelLytic™ MT Cell Lysis Reagent (Sigma) supplemented with protease and phosphatase cocktail inhibitors (Thermo Fisher Scientific). Protein concentration in cell lysates was evaluated using the Bradford method. Equal amounts of protein for each sample were subjected to SDS-PAGE. After electrophoresis, proteins were transferred onto nitrocellulose membranes, and the membranes were subsequently blocked with intercept blocking buffer (Li-Cor) for 1 h at room temperature. Membranes were then incubated overnight at 4°C with primary antibodies against Platelet-Derived Growth Factor BB (PDGF BB, Invitrogen, # BS-1316R, 1:1000), Basic Fibroblast Growth Factor (FGF 2, OriGene, #TA500012, 1:1000); Transforming Growth Factor β 3 (TGFβ3, Abcam, #ab15537, 1:500) β-actin (Cell Signalling, #12262,1:10000), followed by incubation with IRDye® 800CW or 680CW secondary antibody at room temperature for 1 h. The signals were visualized using Odyssey CLx Imaging System (Li-Cor). Densitometry analysis was conducted using Image Studio 5.2. (Li-Cor).

**Statistics.** All values are expressed as mean ± SD. The statistical significance of differences between the two groups was determined using the Mann-Whitney U test for continuous variables that failed Shiparo-Wilk normality tests such as mRNA expression, protein levels, staining quantifications, and functional assays. In blood perfusion curve data, the two groups were tested using multiple Mann-Whitney U tests with Benjamini Krieger and Yekutieli’s adjustment (8). The significant differences that came from *post hoc* comparisons of groups were noted. For the *in vivo* wound closure data, two-way repeated-measures ANOVA followed by *Bonferroni post-hot* testing was used to compare both differences between treatments and time courses (4). In all tests, a value of p < 0.05 was considered statistically significant. The statistical analyses were performed using GraphPad Prism 9 (GraphPad Software).

**Supplemental Figure S1.** **Real-time PCR analysis of inflammatory cytokines in XBP1s-expresisng cells.**


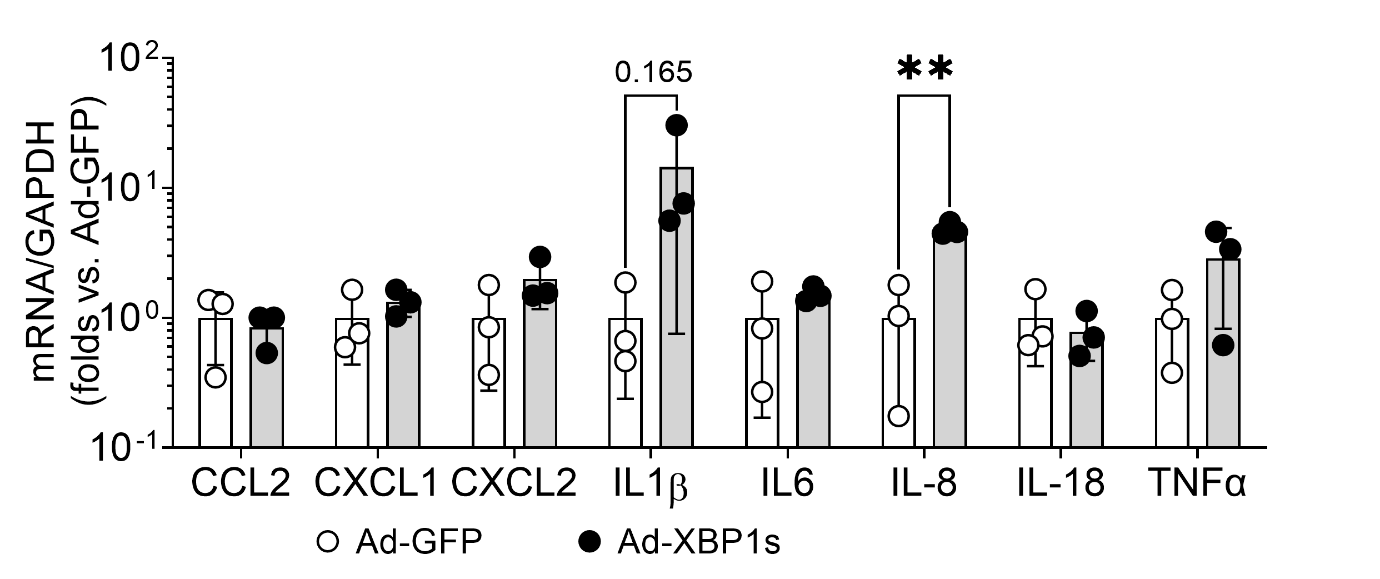


The mRNAs coding growth factors in human embryonic kidney epithelial/fibroblast cells expressing XBP1s or GFP controls. n = 3 per group. *p <0.05.

**SI References**

1. Zhang K*, et al.* (2011) The unfolded protein response transducer IRE1alpha prevents ER stress-induced hepatic steatosis. *EMBO J* 30(7):1357-1375.

2. Soto-Pantoja DR*, et al.* (2014) Thrombospondin-1 and CD47 signaling regulate healing of thermal injury in mice. *Matrix Biol* 37:25-34.

3. Park SW*, et al.* (2014) BRD7 regulates XBP1s' activity and glucose homeostasis through its interaction with the regulatory subunits of PI3K. *Cell Metab* 20(1):73-84.

4. Wang JM*, et al.* (2014) MicroRNA miR-27b rescues bone marrow-derived angiogenic cell function and accelerates wound healing in type 2 diabetes mellitus. *Arterioscler Thromb Vasc Biol* 34(1):99-109.

5. Balaji S*, et al.* (2014) Adenoviral-mediated gene transfer of insulin-like growth factor 1 enhances wound healing and induces angiogenesis. *J Surg Res* 190(1):367-377.

6. Kujawa M*, et al.* (2022) MicroRNA-466 and microRNA-200 increase endothelial permeability in hyperglycemia by targeting Claudin-5. *Molecular therapy. Nucleic acids* 29:259-271.

7. Li H*, et al.* (2021) Novel Role of GPR35 (G-Protein-Coupled Receptor 35) in the Regulation of Endothelial Cell Function and Blood Pressure. *Hypertension* 78(3):816-830.

8. Benjamini Y, Krieger AM, & Yekutieli D (2006) Adaptive linear step-up procedures that control the false discovery rate. *Biometrika* 93(3):491-507.
